# Interactions between *Drosophila* and its natural yeast symbionts—Is *Saccharomyces cerevisiae* a good model for studying the fly-yeast relationship?

**DOI:** 10.7717/peerj.1116

**Published:** 2015-08-25

**Authors:** Don Hoang, Artyom Kopp, James Angus Chandler

**Affiliations:** 1 Department of Evolution and Ecology and Center for Population Biology, University of California, Davis, CA, USA; 2 Current affiliation: Program in Genomics of Differentiation, NIH/NICHD, Bethesda, MD, USA; 3 Current affiliation: Department of Molecular and Cellular Biology, University of California, Berkeley, CA, USA

**Keywords:** *Drosophila melanogaster*, *Saccharomyces cerevisiae*, Baker’s yeast, Yeast, Host-microbe interactions, Microbiome, Microbiota, Symbiosis

## Abstract

Yeasts play an important role in the biology of the fruit fly, *Drosophila melanogaster*. In addition to being a valuable source of nutrition, yeasts affect *D. melanogaster* behavior and interact with the host immune system. Most experiments investigating the role of yeasts in *D. melanogaster* biology use the baker’s yeast, *Saccharomyces cerevisiae*. However, *S. cerevisiae* is rarely found with natural populations of *D. melanogaster* or other *Drosophila* species. Moreover, the strain of *S. cerevisiae* used most often in *D. melanogaster* experiments is a commercially and industrially important strain that, to the best of our knowledge, was not isolated from flies. Since disrupting natural host–microbe interactions can have profound effects on host biology, the results from *D. melanogaster*–*S. cerevisiae* laboratory experiments may not be fully representative of host–microbe interactions in nature. In this study, we explore the *D. melanogaster*-yeast relationship using five different strains of yeast that were isolated from wild *Drosophila* populations. Ingested live yeasts have variable persistence in the *D. melanogaster* gastrointestinal tract. For example, *Hanseniaspora occidentalis* persists relative to *S. cerevisiae*, while *Brettanomyces naardenensis* is removed. Despite these differences in persistence relative to *S. cerevisiae*, we find that all yeasts decrease in total abundance over time. Reactive oxygen species (ROS) are an important component of the *D. melanogaster* anti-microbial response and can inhibit *S. cerevisiae* growth in the intestine. To determine if sensitivity to ROS explains the differences in yeast persistence, we measured yeast growth in the presence and absence of hydrogen peroxide. We find that *B. naardenesis* is completely inhibited by hydrogen peroxide, while *H. occidentalis* is not, which is consistent with yeast sensitivity to ROS affecting persistence within the *D. melanogaster* gastrointestinal tract. We also compared the feeding preference of *D. melanogaster* when given the choice between a naturally associated yeast and *S. cerevisiae*. We do not find a correlation between preferred yeasts and those that persist in the intestine. Notably, in no instances is *S. cerevisiae* preferred over the naturally associated strains. Overall, our results show that *D. melanogaster*-yeast interactions are more complex than might be revealed in experiments that use only *S. cerevisiae*. We propose that future research utilize other yeasts, and especially those that are naturally associated with *Drosophila*, to more fully understand the role of yeasts in *Drosophila* biology. Since the genetic basis of host–microbe interactions is shared across taxa and since many of these genes are initially discovered in *D. melanogaster*, a more realistic fly-yeast model system will benefit our understanding of host–microbe interactions throughout the animal kingdom.

## Introduction

Microbes are vastly important to an animal’s biology (McFall-Ngai et al., 2013). Animal-associated microbes, collectively called the microbiota, help stimulate immunity, assist in nutrient acquisition, and help maintain host homeostasis (Hooper & Macpherson, 2010; Tremaroli & Bäckhed, 2012; Sommer & Bäckhed, 2013). The fruit fly *Drosophila melanogaster* lends itself well as a model for investigating host–microbe interactions (Broderick & Lemaitre, 2012; Erkosar & Leulier, 2014). With only four families of bacteria (Corby-Harris et al., 2007; Wong, Ng & Douglas, 2011; Chandler et al., 2011; Staubach et al., 2013) and typically one family of yeast comprising the majority of taxa associated with *D. melanogaster* (Phaff & Knapp, 1956; Hamby et al., 2012; Chandler, Eisen & Kopp, 2012), the microbiota of *D. melanogaster* is relatively simple compared to that of other animals such as vertebrates (Ley et al., 2008). Furthermore, many members of the *D. melanogaster* microbiota can be cultured using standard media under aerobic conditions, which facilitates experimental studies that associate specific microbes with their hosts (Newell et al., 2014; Chaston, Newell & Douglas, 2014). Combine this simple and culturable microbiota with well-developed genetic tools and well-described immunity pathways, such as the NF-*κ*B, Toll, and Imd pathways (Lemaitre & Hoffmann, 2007), and *D. melanogaster* is a powerful model to understand host–microbe interactions.

Recent work shows the importance of bacteria to *Drosophila*. Larvae raised in the absence of bacteria (i.e., axenically) develop more slowly (Newell et al., 2014) and axenic adults have a reduced lifespan (Brummel et al., 2004; Ridley et al., 2012). Bacteria may also affect mate choice, in that mating is more likely between individuals with more similar bacterial communities (Sharon et al., 2010). Finally, much work has investigated the genetic basis of host interactions with intestinal bacteria (Ryu et al., 2008; Lhocine et al., 2008; Broderick, Buchon & Lemaitre, 2014).

While recent research has generally been focused on bacteria, these microbes are only one taxonomic component of the *D. melanogaster* microbiota. In particular, yeasts are often overlooked during studies of *D. melanogaster*-microbe interactions (Broderick & Lemaitre, 2012). Yeasts affect several aspects of *Drosophila* physiology, behavior, and immunity. For example, particular yeast species affect larval development time and influence adult body weight (Anagnostou, Dorsch & Rohlfs, 2010). Additionally, larvae show preference for yeast species that lead to faster development time and increased adult body weight (Anagnostou, Dorsch & Rohlfs, 2010). Both adults and larvae can influence yeast communities on bananas by reducing yeast species diversity and consistently creating yeast communities of the same restricted set of species (Stamps et al., 2012). Finally, yeast spores can survive digestion by *D. melanogaster*, which suggests that flies can serve as effective vectors of yeasts under natural conditions (Reuter, Bell & Greig, 2007; Coluccio et al., 2008).

Much of the *Drosophila* immune system is devoted to both recognizing and responding to infections by fungi (Lemaitre, Reichhart & Hoffmann, 1997). In particular, the DUOX system (dual oxidases that secrete controlled amounts of reactive oxygen species (ROS)) has been shown to be important in regulating yeasts in the *Drosophila* intestine (Ha et al., 2009). For example, when wild-type flies are fed live *Saccharomyces cerevisiae* (baker’s yeast), the abundance of this yeast rises in the fly intestine initially, but declines to pre-feeding levels (i.e., essentially undetectable) after 24 h. This is in agreement with a classic study investigating the survival of *S. cerevisiae* through the *D. pseudoobscura* digestive tract (Shihata & Mrak, 1951). However, in *D. melanogaster* defective for the DUOX pathway, *S. cerevisiae* continues to rise in abundance, eventually leading to host pathology and increased morality (Ha et al., 2009).

One caveat with the Ha et al. (2009) results (and much of the other work involving *D. melanogaster*-yeast interactions) is that *S. cerevisiae* is rarely found with natural populations of *D. melanogaster* or other *Drosophila* species (Phaff & Knapp, 1956; Lachance, Starmer & Phaff, 1988; Hamby et al., 2012; Chandler, Eisen & Kopp, 2012; Christiaens et al., 2014). While Chandler, Eisen & Kopp (2012) identified sequences related to *S. cerevisiae*, it is unlikely that these sequences represent baker’s yeast in the strict sense. The identified sequences were nearly equally related to *S. cerevisiae* (neotype strain Y-12632) and *S. paradoxus* (neotype strain Y-17217), with 3% and 4% divergence, respectively, at the 26S ribosomal RNA gene. *S. paradoxus* is the closest non-domesticated relative of *S. cerevisiae* and is often found associated with bark, leaves and soil as well as with various *Drosophila* species (Naumov, Naumova & Sancho, 1996; Naumov et al., 2000; Boynton & Greig, 2014). To our knowledge, only one study has definitively identified *S. cerevisiae* in natural populations of *Drosophila*. This study found that approximately 1% of flies in a New Zealand population of *D. simulans* were associated with *S. cerevisiae* (Buser et al., 2014). It should be noted that the unnatural environment of these flies, which were collected in operational vineyards, may be the source of fly-associated *S. cerevisiae* in this case. Moreover, the strain of *S. cerevisiae* most often used in *D. melanogaster*-yeast experiments is a commercially and industrially important strain that, to the best of our knowledge, was not isolated from flies. Since disrupting natural host–microbe interactions can have profound effects on host biology, the results from *D. melanogaster*–*S. cerevisiae* laboratory experiments may not be fully representative of host–microbe interactions as they operate in nature.

Here, we repeat the persistence experiments of Ha et al. (2009) using yeast species that were isolated from *Drosophila* (Phaff & Knapp, 1956; Hamby et al., 2012; Stamps et al., 2012), including *Hanseniaspora uvarum* and *Hanseniaspora occidentalis,* which are commonly associated with natural *Drosophila* populations (Chandler, Eisen & Kopp, 2012). We then attempt to understand the differences in the interactions of *D. melanogaster* with *S. cerevisiae* compared to other yeast species by measuring *Drosophila* feeding preferences and yeast sensitivity to reactive oxygen species.

## Methods

### Yeast strain selection

Yeast strains used in this study are described in Table 1. For *Saccharomyces cerevisiae*, we used Lesaffre instant, which is the same strain as that used in Ha et al. (2009) (W-J Lee, pers. comm., 2012). All yeasts that were isolated from *Drosophila* were obtained from the University of California Phaff Yeast Culture Collection (http://phaffcollection.ucdavis.edu/).

**Table 1:** Yeast strains used in this study.

Yeast species	Abbreviation	Species of *Drosophila* isolated from	Location	Substrate	Phaff yeast collection ID	Reference
*Hanseniaspora occidentalis*	HO	*D. suzukii*	Davis, California, USA	Raspberries	11-1082	Hamby et al. (2012)
*Hanseniaspora uvarum*	HU	*D. suzukii*	Watsonville, California, USA	Raspberries	11-348	Hamby et al. (2012)
*Saccharomyces paradoxus*	SP	*Drosophila* (Obscura group)	Yosemite, California, USA	Unknown^a^	52-153	Phaff & Knapp (1956)
*Brettanomyces naardenensis*	BN	*D. melanogaster*	Davis, California, USA	Banana^b^	09-542	Stamps et al. (2012)
*Debaryomyces hansenii*	DH	*D. melanogaster*	Davis, California, USA	Banana^b^	09-374	Stamps et al. (2012)

**Notes.**

a Flies collected by means of sterile baits, therefore it cannot be determined the substrate most recently visited by the flies.

b Laboratory experiment.

### Yeast persistence

**Figure 1:**
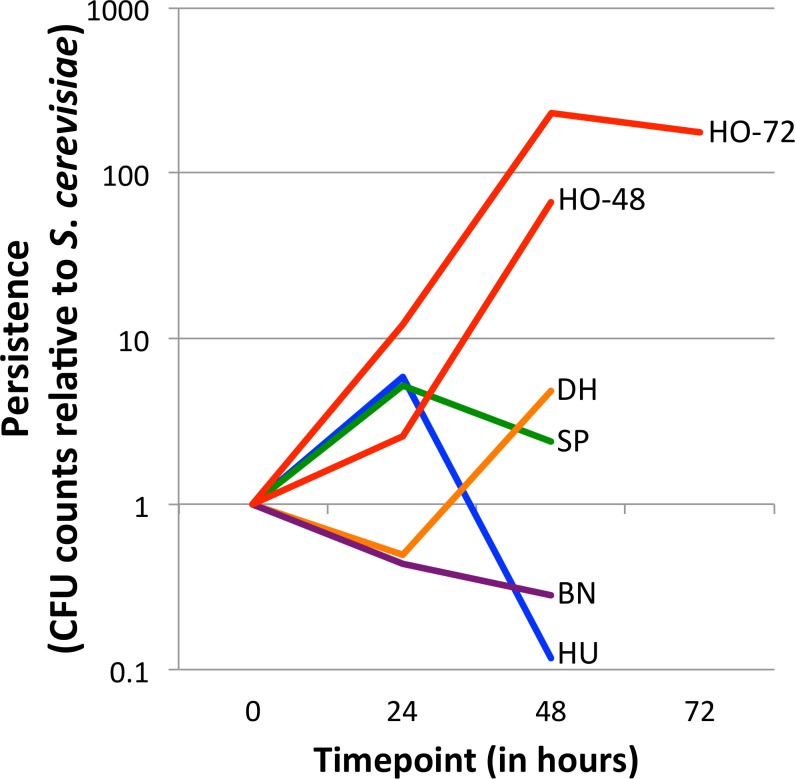
Persistence of yeasts in the *D. melanogaster* intestine relative to *Saccharomyces cerevisiae*. The ratio of the test yeast and *S. cerevisiae* was normalized to 1 at time 0, except for HO-48, which had unusable data for time 0 (see ‘Methods’ section). Values greater than 1 at later timepoints indicate that the test yeast persists relative to *S. cerevisiae*, whereas values less than 1 at later timepoints indicate that the test yeast is removed relative to *S. cerevisiae* (See Eq. (1)). Note the *Y*-axis is log_10_ transformed. Separate graphs for each species, with confidence intervals included, can be found in Fig. S2. HO-48 and HO-72, separate *H. occidentalis* experiments run for 48 and 72 h, respectively; HU, *H. uvarum*; SP, *S. paradoxus*; BN, *B. naardenesis*; DH, *D. hansenii.*

In this experiment, we measured persistence by feeding live yeasts to flies and then measuring how long live yeast colonies could be recovered from dissected gastrointestinal tracts (following (Ha et al., 2009)). At 24–36 h prior to the start of the experiment, adult *D. melanogaster* (3–4 days old, approximately 20 of each sex, isoline 755 (Stamps et al., 2005; Stamps et al., 2012)) were anesthetized under CO_2_ and placed into nine vials containing modified Bloomington media (recipe available in Article S1), (a timeline of the procedure is available as Fig. S1). Two hours prior to the start of the experiment, the flies were starved in empty and autoclaved glass vials. One hour prior to the start of the experiment, the flies were transferred to treatment vials that contained either a confluent growth of *S. cerevisiae* on YPD media (0.5% yeast extract, 1% peptone, 1% dextrose, 2% agar), a confluent growth of the test yeast on YPD media, or a negative control of YPD media only (three replicate vials of each of these three treatments). Immediately after this one hour feeding treatment (which is considered the start of the experiment or time 0), (Fig. S1) flies were transferred into vials containing sterile YPD media, with additional transfers to fresh, sterile YPD containing vials every 12 h. At 0 h, 24 h, and 48 h (and, where applicable, 72 h), five male and five female flies from each vial had their entire gastrointestinal tracts including crops dissected out. Since an early experiment with *H. occidentalis* suggested it persisted relative to *S. cerevisiae* at 24 and 48 h (see below and Fig. 1 and Table 2), we performed an additional experiment with *H. occidentalis* to 72 h. While we did not explicitly measure fly phenotypic responses to different yeasts (except for feeding preference, see below), there were no conspicuous effects on fly survival or behavior. These 10 gastrointestinal tracts were pooled into one sample. Then, each sample was homogenized, and put through a serial dilution from 1 to 1/1,000 times the original concentration. 10 µL of each 200 µL dilution of each sample were plated onto yeast-selective Rose Bengal Chloramphenicol Agar plates (Fisher Scientific Catalog #OXCM0549B). The number of colony forming units (CFUs) was determined for each treatment and replicate. CFUs shall henceforth be used as a measure of total yeast abundance within the flies. Any experiments with greater than 50 CFUs per fly in the negative control at time zero were discarded (average number of CFUs in the *S. cerevisiae* treatments was more than 200,000 CFUs per fly). The most concentrated dilution for which individual CFUs were visible (i.e., were not confluent) was used for analysis. All raw data for the persistence experiments is available in Data S1.

**Table 2:** Persistence of yeasts in the *D. melanogaster* gastrointestinal tract relative to *Saccharomyces cerevisiae*. The ratio of the test yeast and *S. cerevisiae* was normalized to 1 at time 0, except for HO-48, which had unusable data at time 0 (see ‘Methods’ section). Values greater than 1 at later timepoints indicated that the test yeast persists relative to *S. cerevisiae*, whereas values less than 1 at later timepoints indicate that the test yeast is removed relative to *S. cerevisiae* (See Eq. (1)). Significance was then determined by whether the 95%, 99%, or 99.9% confidence intervals overlap 1.

Time	HO-48	HO-72	HU	SP	BN	DH
24	Persists at 99%	Persists at 99.9%	No change	Persists at 95%	Removed at 99.9%	Removed at 99.9%
48	Persists at 99.9%	Persists at 95%	Removed at 99.9%	No change	Removed at 99.9%	Persists at 99.9%
72		Persists at 99.9%				

**Notes.**

HO-48 and HO-72 separate *H. occidentalis* experiments run for 48 and 72 h, respectively HU
*H. uvarum*
SP
*S. paradoxus*
BN
*B. naardenesis*
DH
*D. hansenii*

For each experiment, relative persistence was determined by the ratio of the test yeast compared to *S. cerevisiae* at the 24, 48, and (in one experiment) the 72 h time points (Fig. 1 and Fig. S2), (Eq. (1)). To account for potential differences in initial uptake, these ratios are normalized to the ratio of test yeast to *S. cerevisiae* at time 0 (Eq. (1)).

(1) \begin{eqnarray*} \frac{T e s t \hspace{0.167em} y e a s t \hspace{0.167em} (R e p l i c a t e \hspace{0.167em} X \hspace{0.167em} a t \hspace{0.167em} 24,48,\hspace{0.167em} o r \hspace{0.167em} 72 \hspace{0.167em} \mathrm{h})}{S C \hspace{0.167em} (A v e r a g e \hspace{0.167em} o f \hspace{0.167em} 3 \hspace{0.167em} r e p l i c a t e s \hspace{0.167em} a t \hspace{0.167em} 24,48 \hspace{0.167em} o r \hspace{0.167em} 72 \hspace{0.167em} \hspace{0.167em} \mathrm{h})}/\frac{T e s t \hspace{0.167em} y e a s t \hspace{0.167em} (R e p l i c a t e \hspace{0.167em} X \hspace{0.167em} a t \hspace{0.167em} 0 \hspace{0.167em} \mathrm{h})}{S C \hspace{0.167em} (A v e r a g e \hspace{0.167em} o f \hspace{0.167em} 3 \hspace{0.167em} r e p l i c a t e s \hspace{0.167em} a t \hspace{0.167em} \mathrm{0~ h})}. \end{eqnarray*}

Note that for each experiment there are three replicates for each test yeast and *S. cerevisiae* (henceforth referred to as TY and SC, respectively). The TY and SC replicates are not paired (that is TY1 is not related to SC1, and TY2 is not related to SC2, and so forth) and therefore the average of the SC replicates are taken as the denominators in Eq. (1). In one early experiment for which normalization was not possible (CFU counts at time 0 were so high for both TY and SC that a “lawn” was present; no dilutions were performed; experiment HO-48), a normalization factor of 1:1 was used. The 95%, 99% and 99.9% confidence intervals of relative persistence estimates were determined for each yeast and time point. Whether these intervals overlap one, which signifies no persistence change relative to SC, is summarized in Table 2. Finally, the ratio of absolute cell counts (CFUs) between each 24-hour period is shown in Table 3 (Raw data is available in Data S1).

**Table 3:** Clearance of yeasts from the *D. melanogaster* gastrointestinal tract. The values measure the ratio of the absolute number of colony forming units (CFUs) per fly for each timeframe. A value of 1 would indicate that the CFUs remain unchanged, whereas a value of 10 would indicate that there were 10 times fewer CFUs at the later time point. Note that for all yeasts and all timeframes, the number of CFUs decreased over time.

	HO-48	HO-72	HU	SP	BN	DH	Average^a^	SC average^b^
0–24	N/A^c^	7.9	11.2	24.0	7.6	23.3	14.8	64.5
24–48	10.4	3.5	650.0	266.1	4028.6	88.9	841.3	776.5

**Notes.**

HO-48 and HO-72 separate *H. occidentalis* experiments run for 48 and 72 h, respectively HU
*H. uvarum*
SP
*S. paradoxus*
BN
*B. naardenesis*
DH
*D. hansenii*

a Average for all strains, but not including *S. cerevisiae*.

b Average for the *S. cerevisiae* controls in all experiments.

c No data for 0 timepoint.

### Yeast sensitivity to reactive oxygen species (ROS)

**Figure 2:**
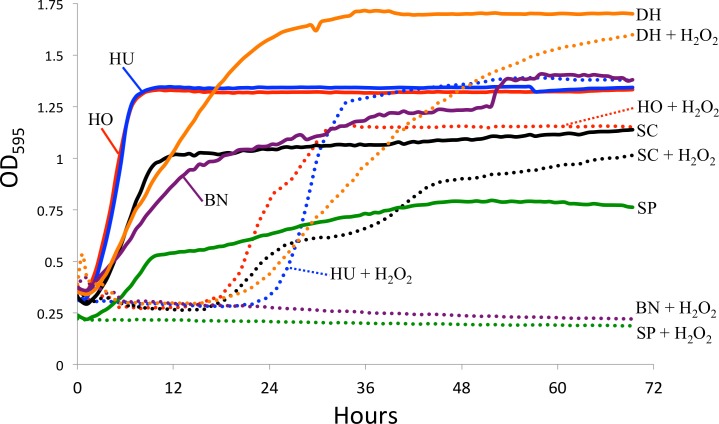
Yeast growth *in vitro*. Yeast growth as measured by optical density in a TECAN spectrophotometer. Hydrogen peroxide (H_2_O_2_) was added to mimic reactive oxygen species in the *D. melanogaster* intestine. The control curves (i.e., without H_2_O_2_) are drawn as solid lines and the H_2_O_2_ treatment as dotted lines. SC, *S. cerevisiae*; HO, *H. occidentalis*; HU, *H. uvarum*; SP, *S. paradoxus*; BN, *B. naardenesis*; DH, *D. hansenii.*

Since intestinal reactive oxygen species are one potential factor limiting *in vivo* yeast growth (Ha et al., 2009), we tested yeast sensitivity to hydrogen peroxide (a generator of ROS) *in vitro*. Specifically, we measured yeast growth in the absence and presence of 0.5 mM hydrogen peroxide (H_2_O_2_). 1 mM H_2_O_2_ leads to approximately 50% survival in *S. cerevisiae* (Jamieson, 1992). One colony of each yeast species was added to individual glass test tubes containing four mL of liquid YPD media and shaken at 27 °C overnight. The following day, three replicates of each yeast was added into a 96 well plate. Each well contained 150 µL of liquid YPD media, 10 µL of mineral oil (to limit evaporation), and 10 µL of the liquid yeast culture. For the experiments testing ROS resistance, an additional 2 µL of H_2_O_2_ was added to each well, creating a final concentration of 0.5 mM H_2_O_2_. Optical density was measured every 30 min for three days using a TECAN spectrophotometer. The average for the three replicates is shown in Fig. 2. Because we did not standardize the amount of yeast cells at the start of the experiment, we are limiting our conclusions to those within strains, specifically the effect of H_2_O_2_ on growth. All raw data for the yeast growth experiments is available in Data S2.

### Feeding preference

To measure feeding preference, flies were allowed access to the test yeast and *S. cerevisiae* that were labeled with either blue or red food dyes and then coloration of the fly abdomens was scored (methods adapted from Tanimura et al., 1982; Weiss et al., 2011). 100 mm × 15 mm sized Petri plates of 1% agar were prepared and had two holes punched out using the large end of a 1,000 µL pipette tip. These holes were then filled with YPD agar media. On each YPD core, a liquid solution of either *S. cerevisiae* or the test yeast was added. 48 h later, 8 µL of either blue or red food dye (McCormick and Company Inc.) was added the patches of yeast. To control for potential preference for the dye itself, both dye combinations (i.e., TY = blue/SC = red and TY = red/SC = blue) were used. Furthermore, no-choice controls (i.e., SC = red/SC = blue and TY = red/TY = blue) were also included, resulting in a total of four treatments per experiment. Three or four replicates were performed for each treatment (further experimental details available in Article S2).

Approximately 50 3–4 day old *D. melanogaster* adults were added to each plate after first being starved for an hour as in the persistence experiments. The flies were allowed to feed for approximately 1.5 h in the dark and then their abdomens were scored, after the scorer was blinded to the treatments, for 4 categories: blue, red, purple or empty abdomens.

**Figure 3:**
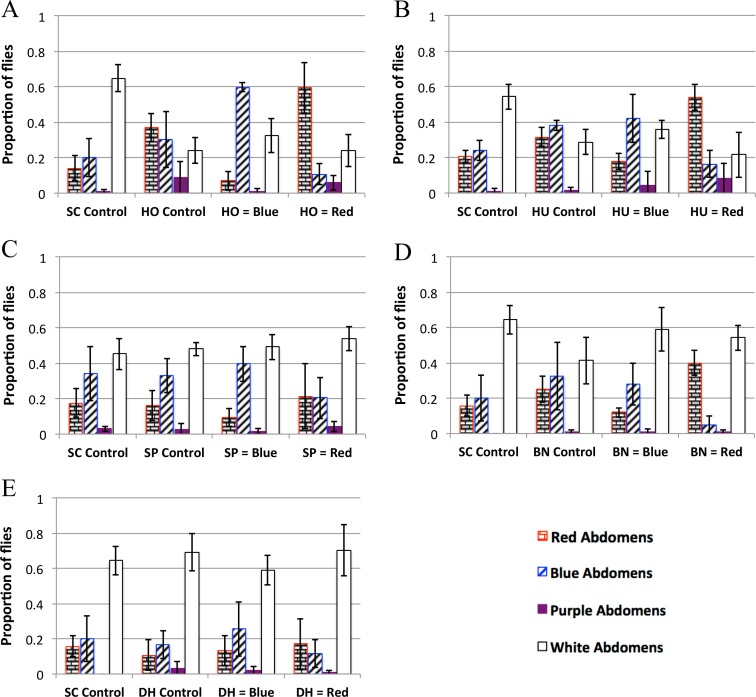
Yeast feeding preference of *D. melanogaster*. Feeding preference as measured by scoring abdomen color after simultaneous access to two yeast cultures, one labeled with red dye and the other labeled with blue dye. In the controls, both cultures of yeast were the same (e.g., one culture of blue labeled *S. cerevisiae* and one culture of red *S. cerevisiae*). For each experiment in each panel the first column is the proportion of flies with red abdomens (rectangular brick pattern); second column: proportion of flies with blue abdomens (diagonal lines); third column: proportion of flies with purple abdomens (solid color); fourth column: proportion of white (i.e., no-color) abdomens (no-fill). Experiments 3D and 3E were done concurrently and therefore have the same SC control. 3A: *H. occidentalis*. 3B: *H. uvarum*. 3C: *S. paradoxus*. 3D: *B. naardenesis*. 3E: *D. hansenii*. Error bars represent ±1 SD.

For each experiment, the proportion of flies with each abdomen color in each replicate is shown in Fig. 3. Preference was calculated by first taking the average of each set of replicates within a treatment/experiment and then applying an established Preference Index (Dus et al., 2011), (Eq. (2)).

(2) \begin{eqnarray*} \frac{F l i e s \hspace{0.167em} w i t h \hspace{0.167em} c o l o r e d \hspace{0.167em} a b d o m e n s \hspace{0.167em} \left(e i t h e r \hspace{0.167em} r e d \hspace{0.167em} o r \hspace{0.167em} b l u e\right)\hspace{0.167em} +\hspace{0.167em} \frac{F l i e s \hspace{0.167em} w i t h \hspace{0.167em} p u r p l e \hspace{0.167em} a b d o m e n s}{2}}{T o t a l \hspace{0.167em} f l i e s \hspace{0.167em} t h a t \hspace{0.167em} f e d}. \end{eqnarray*}

Since a PI of 0.5 indicates no preference for either color, significance was determined by whether the 95%, 99% and 99.9% confidence intervals overlap 0.5 (Table 4 and Fig. S3). All raw data for the yeast growth experiments is available in Data S3.

**Table 4:** Yeast feeding preference of *D. melanogaster*. Significance is determined by whether the 95%, 99%, or 99.9% confidence intervals overlap 0.5, which indicates equal ingestion of both the red and blue labeled yeasts (Eq. (2)).

	SC control	Test yeast control	Test yeast is labeled blue	Test yeast is labeled red
HO	No preference	No preference	Prefers HO at 99.9%	Prefers HO at 99.9%
HU	No preference	Prefers blue at 95%	Prefers HU at 99%	Prefers HU at 99.9%
SP	No preference	No preference	Prefers SP at 99.9%	No preference
BN	No preference^a^	No preference	Prefers BN at 99%	Prefers BN at 99.9%
DH	No preference^a^	No preference	No preference	No preference

**Notes.**

SC
*S. cerevisiae*
HO
*H. occidentalis*
HU
*H. uvarum*
SP
*S. paradoxus*
BN
*B. naardenesis*
DH
*D. hansenii*

a These two experiments were done concurrently and therefore used the same SC control.

## Results and Discussion

Yeast strains naturally associated with *Drosophila* differ in their ability to persist in the *D. melanogaster* gastrointestinal tract (Fig. 1, Fig. S2 and Table 2). Overall, there is no trend for persistence between the naturally associated yeasts and *S. cerevisiae*: One yeast persists at all time points (*H. occidentalis*), one yeast is removed at all time points (*B. naardenensis*), and the remaining yeasts are not consistent at different time points. Together, these results show that yeast persistence in the *D. melanogaster* gastrointestinal tract is more variable than would be suggested by *S. cerevisiae* studies alone.

Our results find that *H. occidentalis* persists relative to *S. cerevisiae* in the *D. melanogaster* intestinal tract. Furthermore, this yeast retained moderate counts (CFUs) per fly even at the final time point. At 72 h, 9,327 CFUs are present per fly for *H. occidentalis* compared to 3 CFUs for *S. cerevisiae* (Data S1). The average for all strains, excluding *H. occidentalis* but including *S. cerevisiae*, at 48 h is 98 CFUs (Data S1). Given that ingestion to excretion transit time for actively feeding flies can be under one hour (Wong et al., 2008), it is unlikely that individual *H. occidentalis* cells from the feeding treatment were retained until the final timepoint. Furthermore, since our methods transferred flies to sterile media every twelve hours, live yeasts would likely not be re-ingested in high amounts. However, our raw colony counts find that all yeasts decline in abundance over time (Data S1). On average for the test yeasts (i.e., not *S. cerevisiae*), there are 15 times fewer viable cells from the start of the experiment to 24 h and 841 times fewer between 24 h and 48 h (Table 3). Even *H. occidentalis* declines in total abundance, though much less quickly than the other yeasts between 24 and 48 h (Table 3). Taken together, it remains unknown if any yeasts establish stable populations within the *D. melanogaster* gastrointestinal tract. A similar scenario occurs with *D. melanogaster*-associated bacteria, which require frequent replenishment (by ingestion of bacteria-contaminated media) to maintain high intra-fly bacterial abundances (Blum et al., 2013).

There are several ways in which microbes may persist within their hosts. The first involves forming biofilms to anchor themselves to their host, as occurs with the bacterial symbionts of bean bugs (Kim et al., 2014) and squids (Chavez-Dozal et al., 2012) and the opportunistic human pathogen, the yeast *Candida albicans* (Mathé & Van Dijck, 2013). It is unknown if yeasts form biofilms in *D. melanogaster*, but this could be investigated by staining for an extracellular polysaccharide matrix within the intestinal lumen (Kim et al., 2014).

Another explanation for the differences in yeast persistence may be variable growth rates in the *D. melanogaster* gastrointestinal tract and, in particular, variable sensitivity to the reactive oxygen species (ROS) that are produced in the *D. melanogaster* intestine in response to live yeasts (Ha et al., 2009). We therefore measured the *in vitro* growth rates of these yeasts both in the absence and in the presence of ROS-producing hydrogen peroxide (H_2_O_2_), (Fig. 2). Since we did not standardize the absolute number of cells inoculated for each yeast, the timing of the beginning of exponential growth between treatments is not comparable between yeasts. However, since the same inoculum was used for each yeast regardless of treatment, we can examine the effect of H_2_O_2_ on the growth of each yeast species. In stark contrast with all other yeasts, *S. paradoxus* and *B. naardenensis* do not show any growth when exposed to H_2_O_2_ (Fig. 2). One of these *(B. naardenesis)* is the only strain that is removed, relative to *S. cerevisiae*, at all time points (Fig. 1 and Table 2). Conversely, *H. occidentalis,* which is the only strain that persists at all time points, can still grow after exposure to H_2_O_2_. Together, this is consistent with yeast sensitivity to ROS affecting persistence within the *D. melanogaster* gastrointestinal tract. Interestingly, ROS have been proposed as a fundamental factor structuring host–microbe interactions (Moné, Monnin & Kremer, 2014). However, other factors, such as sensitivity to antimicrobial peptides (AMPs), are likely also important. Future studies could investigate the relative roles of ROS production and AMPs in affecting gastrointestinal persistence.

The feeding behavior data show that *D. melanogaster* prefers three yeasts (*H. occidentalis*, *H. uvarum*, and *B. naardenensis*) and shows no preference for one yeast (*D. hansenii*), (Fig. 3, Fig. S3, and Table 4). In one case (*S. paradoxus*), the naturally associated yeast was preferred when labeled blue, but not when labeled red. Notably, in no instances was *S. cerevisiae* preferred. Furthermore, in control experiments where *S. cerevisiae* was the only option, fewer flies fed overall than in control experiments where a preferred test yeast was the only choice (white bars, Figs. 3A, 3B, and 3D). These results are likely not due to flies having access to fewer overall cells of *S. cerevisiae* compared to the other yeasts because the number of yeast cells did not seem to be a limiting factor during the feeding treatment. In particular, the colonies of growing yeasts were much larger than the flies themselves (Article S2) and the colonies were never fully consumed by the end of the feeding treatment (data not shown).

Since the strain of *S. cerevisiae* used here was not (to our knowledge) isolated from *Drosophila* in nature, overall our findings suggest that naturally occurring yeasts are generally more preferable. Similar results find that *D. simulans* is more attracted to *S. cerevisiae* strains that were naturally associated with *D. simulans* when compared to a panel of 92 other strains of *S. cerevisiae* (Buser et al., 2014). Furthermore, yeasts that were isolated from fruit associated environments were more attractive than yeasts isolated from other environments (Palanca et al., 2013). Overall, these studies suggest a general positive preference/attractiveness towards naturally associated yeast strains (but see a discussion of feeding preference compared to attractiveness below).

We do not find a correlation between feeding preference and gastrointestinal persistence. In particular, the yeasts that are preferred (*H. occidentalis*, *H. uvarum*, and *B. naardenesis*) include both a yeast that persist at all time points (*H. occidentalis*) and one that is removed at all time points (*B. naardenesis*). Likewise the yeast that is not preferred (*D. hansenii*) is not removed relative to *S. cerevisiae* at the 48 h timepoint. Taken together, it appears that *D. melanogaster* yeast preference does not explain the differences in persistence identified in this study.

In nearly all the controls, there was no color preference (Table 3 and Fig. S3). These experiments were done in the dark to reduce any potential effects of visual cues on *D. melanogaster* preference. A pilot experiment done in the light found a strong preference for blue relative to red in control treatments (Fig. S4, Raw data found in Data S3). This, along with the results in Table 4 and Fig. 3, suggest that *D. melanogaster* feeding preference involves input from multiple senses, a result that has been found in other insects such as bees (Leonard & Masek, 2014) and mosquitoes (Gibson & Torr, 1999).

Much recent work has explored *D. melanogaster* attraction to different yeast species and genotypes. Most studies did not allow flies to interact directly with the yeast cells or, once an individual fly made a choice (e.g., they entered a tube containing the yeast), they were inhibited from selecting the other choice (Anagnostou, Dorsch & Rohlfs, 2010; Palanca et al., 2013; Schiabor, Quan & Eisen, 2014; Buser et al., 2014; Christiaens et al., 2014). Therefore, the flies were presumably responding to airborne volatile compounds. In the *D. melanogaster*–yeast system, the interaction and relative importance of attractiveness (as measured in these previous studies) and feeding (as measured in the current study) remain unclear for fly behavior under natural conditions.

Finally, we note that not all the yeasts used in this study were isolated from *D. melanogaster* (Table 1) and it remains unknown how differences in host ecology affect the fly-yeast relationship. For example, *D. suzukii* can utilize live undamaged fruit, while members of the *D. obscura* group can utilize sap fluxes. This is distinct from the decomposing fruits that *D. melanogaster* commonly uses as feeding and breeding sites (though *D. suzukii* also uses decomposing fruits, when available, as readily as other species). Future work could explore yeast persistence within the gastrointestinal tracts of their specific hosts and *D. suzukii* and *D. obscura* preferences for these yeasts. Such comparative experiments would help illuminate how yeast species, host species, and host ecology interact to shape the fly-yeast relationship in a natural context.

## Conclusion

Fundamental insights into host–microbe interactions have been gained using *Drosophila melanogaster* as a model organism. At least as early as 1916, researchers have been investigating *D. melanogaster-*yeast interactions (Loeb & Northrop, 1916). During the 1950s, work was done on the attraction of flies to yeasts that were isolated from wild-caught *Drosophila* (Dobzhansky et al., 1956). Unfortunately, many recent studies have used commercial strains of *S. cerevisiae*, as opposed to yeasts that are naturally associated with *Drosophila*. In the current study, we find differences in gastrointestinal persistence and feeding preference between these naturally associated yeasts and *S. cerevisiae*. Whether these yeasts interact differently with the *D. melanogaster* immune system, for example by being less pathogenic than *S. cerevisiae* in immune-deficient flies (Ha et al., 2005; Ha et al., 2009), is unknown. Since the genetic basis of host–microbe interactions is shared across taxa and since many of these genes are initially discovered in *D. melanogaster*, a more realistic fly-yeast model system will benefit our understanding of host–microbe interactions throughout the animal kingdom. We therefore encourage future researchers to incorporate yeast species that are naturally associated with *Drosophila* into their studies.

## Supplemental Information

10.7717/peerj.1116/supp-1 Figure S1 Timeline of the persistence assays

10.7717/peerj.1116/supp-2 Figure S2 Persistence graphs for each yeast strain The ratio of the test yeast and *S. cerevisiae*was normalized to 1 at time 0, except for HO-48, which had unusable data for time 0 (see ‘Methods’ section). Values greater than 1 at later timepoints indicate that the test yeast persists relative to *S. cerevisiae*, whereas values less than 1 at later timepoints indicate that the test yeast is removed relative to *S. cerevisiae.* Error bars represent ± the 95%, 99%, or 99.9% confidence intervals, indicated by one, two, or three asterisks, respectively. Note the scale of the *Y*-axis differs between panels. A combined graph, showing the only the average value for each strain, can be found in Fig. 1. HO-48 and HO-72: Separate *H. occidentalis* experiments run for 48 and 72 h, respectively. HU, *H. uvarum*; SP, *S. paradoxus*; BN, *B. naardenesis*; DH, *D. hansenii*.

10.7717/peerj.1116/supp-3 Figure S3 Preference indices for each feeding experiment Feeding preference as measured by scoring abdomen color after simultaneous access to two yeast cultures, one labeled with red dye and the other labeled with blue dye. In the controls, both cultures of yeast were the same (e.g., one culture of blue labeled *S. cerevisiae* and one culture of red *S. cerevisiae*). The *X*-axis is the preference index (Eq. (2)). A preference index of 1 indicates ingestion of only blue-labeled yeasts, while a preference index of 0 indicates ingestion of only red-labeled yeasts. For each panel, the uppermost point represents the *S. cerevisiae* control (black circle), the second point represents the test yeast control (black square), the third point represents when the test yeast is labeled blue (blue square), and the lowermost point represents when the test yeast is labeled red (red square). Whether the 95%, 99%, or 99.9% confidence intervals overlap 0.5 (which would indicate no preference) is indicated by one, two, or three asterisks, respectively. BN and DH were done concurrently and therefore have the same SC control. HO, *H. occidentalis*; HU, *H. uvarum*; SP, *S. paradoxus*; BN, *B. naardenesis*; DH, *D. hansenii*.

10.7717/peerj.1116/supp-4 Figure S4 Pilot preference experiment This experiment was done by placing the experimental arenas (i.e., petri dishes) under a cardboard box, which was not completely dark inside. Future experiments (Fig. 3 and Fig. S3) were performed inside a well-sealed drawer.

10.7717/peerj.1116/supp-5 Data S1 Raw data for the persistence experiments Values are the number of CFUs per fly after correcting for the dilution factor, the volume plated, and the number of flies per sample.

10.7717/peerj.1116/supp-6 Data S2 Raw data for the yeast growth experiments

10.7717/peerj.1116/supp-7 Data S3 Raw data for the feeding preference experiments

10.7717/peerj.1116/supp-8 Article S1 Modified Bloomington Drosophila Media Recipe

10.7717/peerj.1116/supp-9 Article S2 Protocol for choice feeding assays
