# Supplementary figures and images for: Interactions between Drosophila and its natural yeast symbionts—Is Saccharomyces cerevisiae a good model for studying the fly-yeast relationship?
Source: PeerJ. 2015 Aug 25;3:e1116. doi: 10.7717/peerj.1116 (PMC4556146; doi:10.7717/peerj.1116)

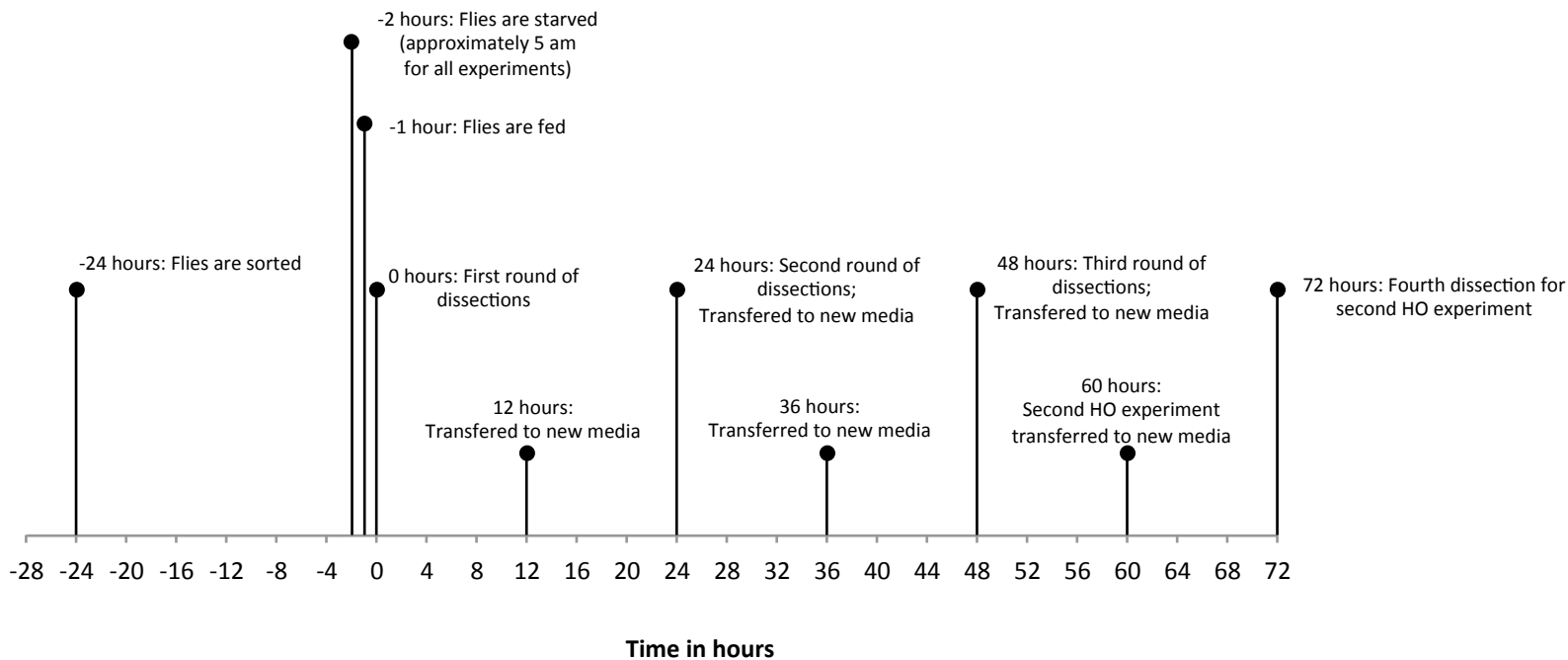

Supplement: Figure S1 [file peerj-03-1116-s001.pdf]

Persistence

(CFU counts relative to *S. cerevisiae*)

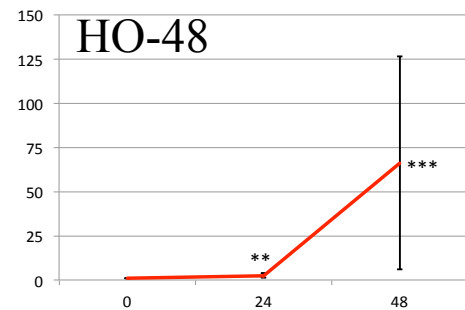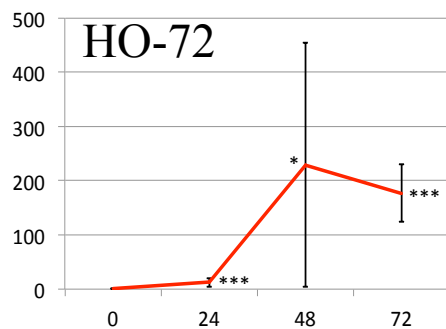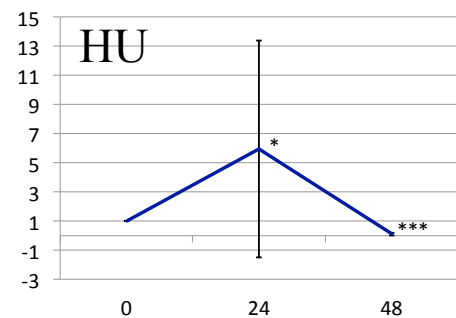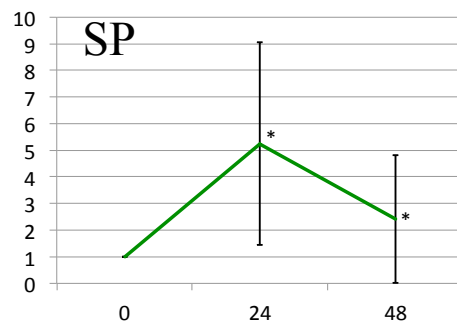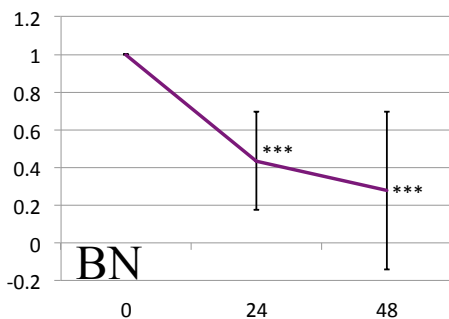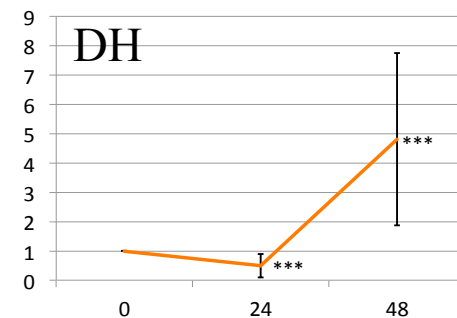

Timepoint (in hours)

Supplement: Figure S2 — The ratio of the test yeast and S. cerevisiaewas normalized to 1 at time 0, except for HO-48, which had unusable data for time 0 (see ‘Methods’ section). Values greater than 1 at later timepoints indicate that the test yeast persists relative to S. cerevisiae, whereas values less than 1 at later timepoints indicate that the test yeast is removed relative to S. cerevisiae. Error bars represent ± the 95%, 99%, or 99.9% confidence intervals, indicated by one, two, or three asterisks, respectively. Note the scale of the Y-axis differs between panels. A combined graph, showing the only the average value for each strain, can be found in Fig. 1. HO-48 and HO-72: Separate H. occidentalis experiments run for 48 and 72 h, respectively. HU, H. uvarum; SP, S. paradoxus; BN, B. naardenesis; DH, D. hansenii. [file peerj-03-1116-s002.pdf]

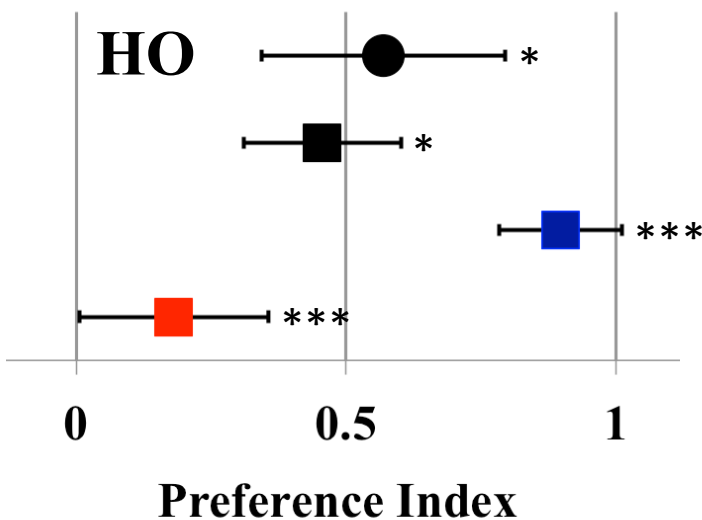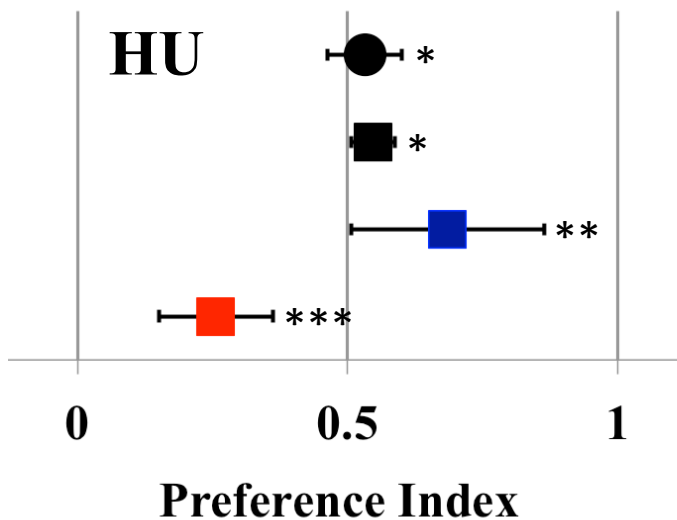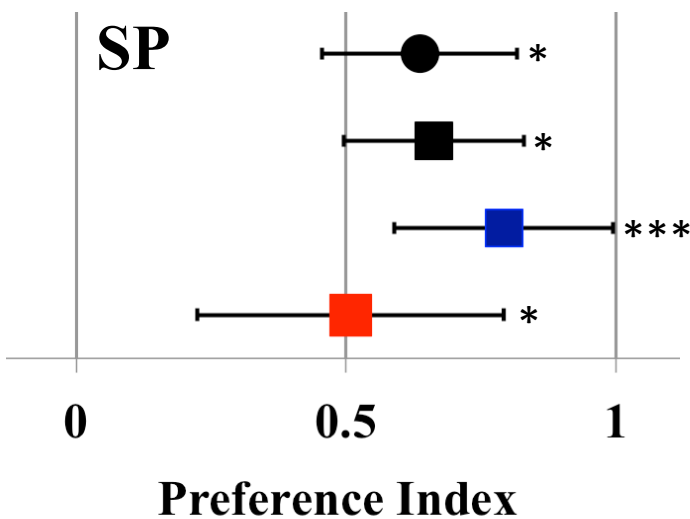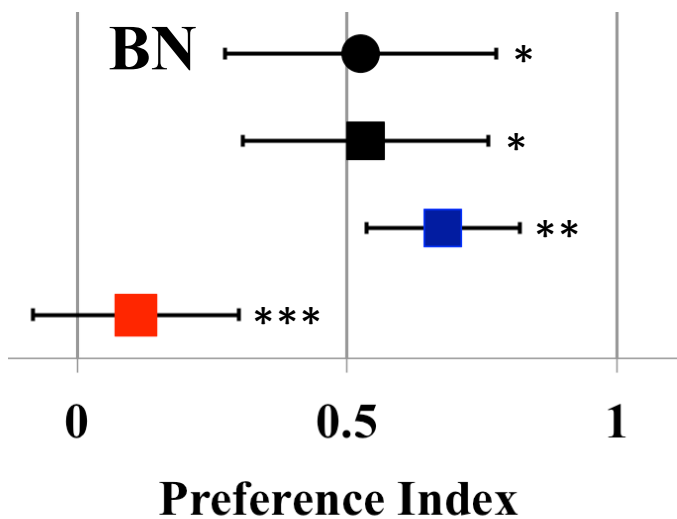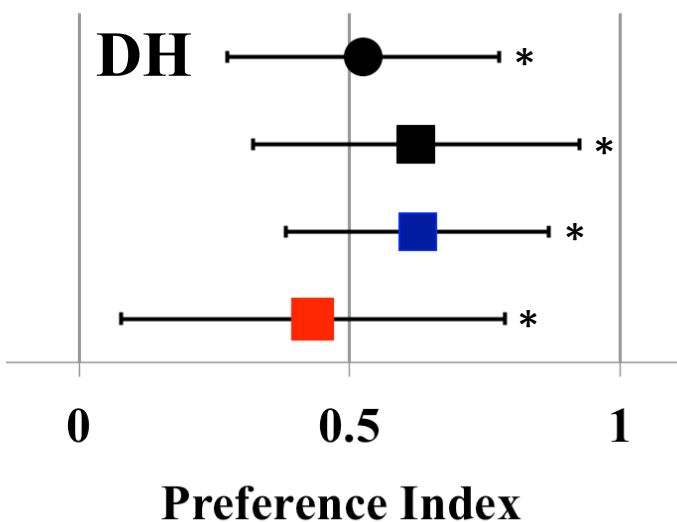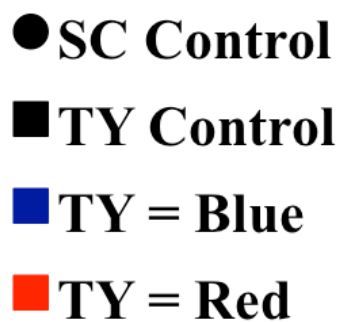

Supplement: Figure S3 — Feeding preference as measured by scoring abdomen color after simultaneous access to two yeast cultures, one labeled with red dye and the other labeled with blue dye. In the controls, both cultures of yeast were the same (e.g., one culture of blue labeled S. cerevisiae and one culture of red S. cerevisiae). The X-axis is the preference index (Eq. (2)). A preference index of 1 indicates ingestion of only blue-labeled yeasts, while a preference index of 0 indicates ingestion of only red-labeled yeasts. For each panel, the uppermost point represents the S. cerevisiae control (black circle), the second point represents the test yeast control (black square), the third point represents when the test yeast is labeled blue (blue square), and the lowermost point represents when the test yeast is labeled red (red square). Whether the 95%, 99%, or 99.9% confidence intervals overlap 0.5 (which would indicate no preference) is indicated by one, two, or three asterisks, respectively. BN and DH were done concurrently and therefore have the same SC control. HO, H. occidentalis; HU, H. uvarum; SP, S. paradoxus; BN, B. naardenesis; DH, D. hansenii. [file peerj-03-1116-s003.pdf]

Proportion of flies

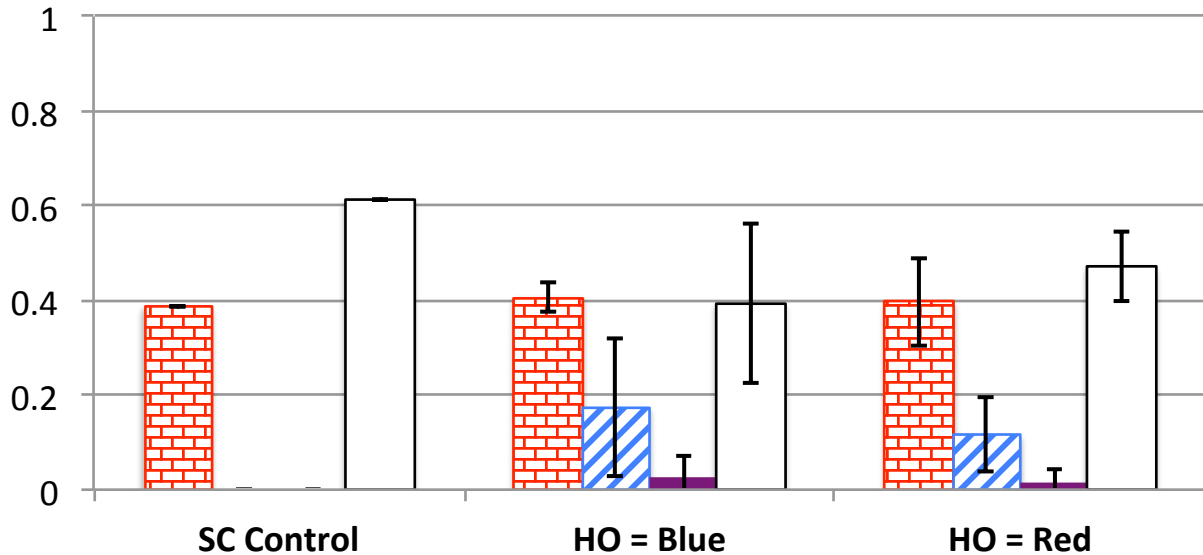

Supplement: Figure S4 — This experiment was done by placing the experimental arenas (i.e., petri dishes) under a cardboard box, which was not completely dark inside. Future experiments (Fig. 3 and Fig. S3) were performed inside a well-sealed drawer. [file peerj-03-1116-s004.pdf]
