# Supplementary material for: Interactions between Drosophila and its natural yeast symbionts—Is Saccharomyces cerevisiae a good model for studying the fly-yeast relationship?
Source: PeerJ. 2015 Aug 25;3:e1116. doi: 10.7717/peerj.1116 (PMC4556146; doi:10.7717/peerj.1116)
Supplement: Article S1 [file peerj-03-1116-s008.docx]

**Modified Bloomington Drosophila Media Recipe**

Total Volume 31 liters

Ingredients:

Agar – 275 milliliters

27 Liters of Hot Water

Non-Active Yeast – 600 milliliters

Soy Flour – 500 milliliters

Corn Meal – 2100 milliliters

Malt Extract – 1475 milliliters

Corn Syrup, Light – 1.5 Liters

Acid Mix A (0.4% Propionic Acid and 0.06% Phosphoric Acid in water) – 315 milliliters

Tegosept Mix (38 grams Tegosept in 138 milliliters ethanol) – 200 milliliters

4 Liters of water is added after the food has come to a boil
