# Supplementary material for: Interactions between Drosophila and its natural yeast symbionts—Is Saccharomyces cerevisiae a good model for studying the fly-yeast relationship?
Source: PeerJ. 2015 Aug 25;3:e1116. doi: 10.7717/peerj.1116 (PMC4556146; doi:10.7717/peerj.1116)
Supplement: Article S2 [file peerj-03-1116-s009.docx]

Step 1: Fill a 10cm Petri dish with 25mL of 1% agar.
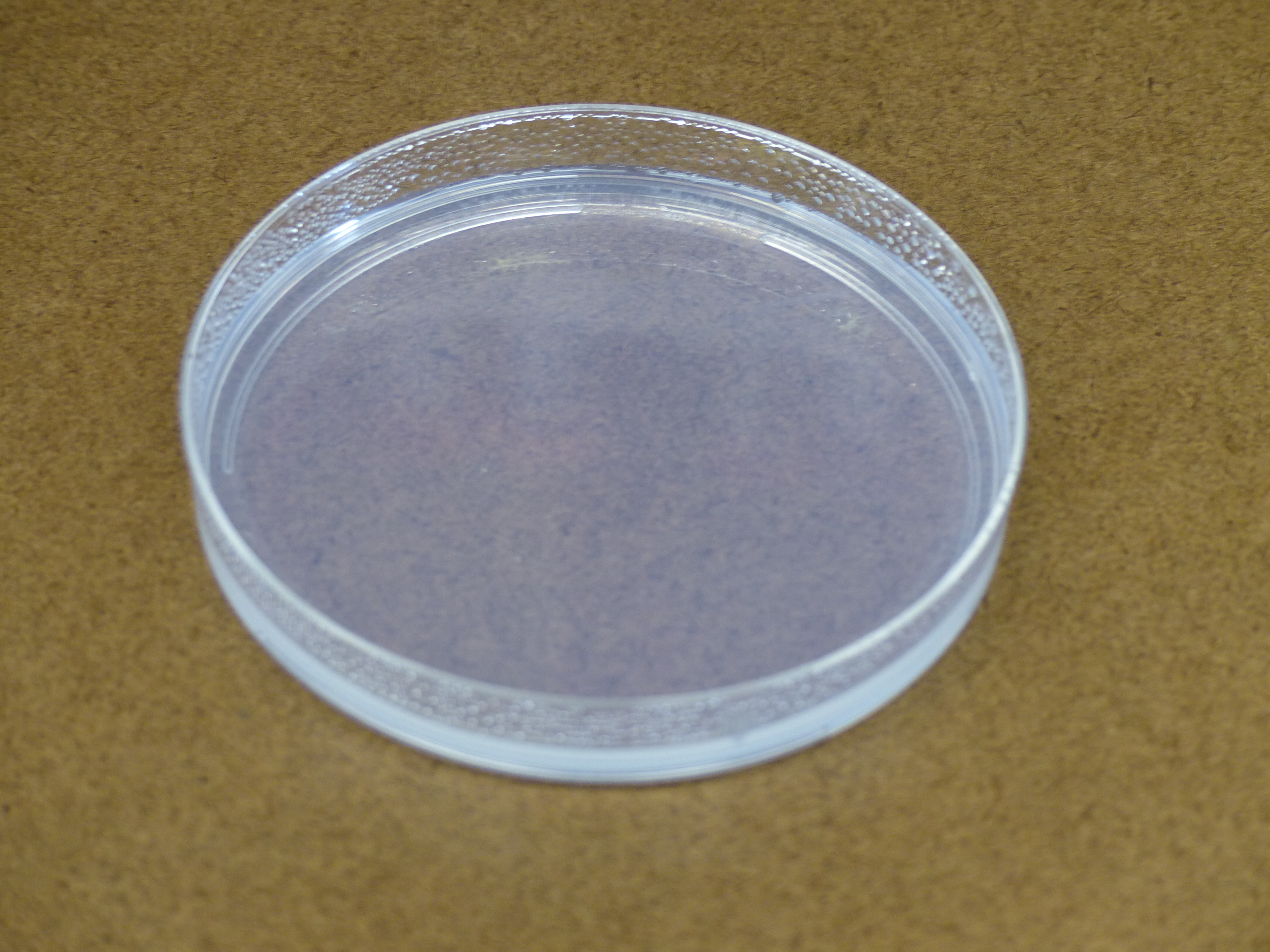


Step 2: Using the large end of a 1000 ml pipet, remove two cores of media from either end of a 1% agar plate.


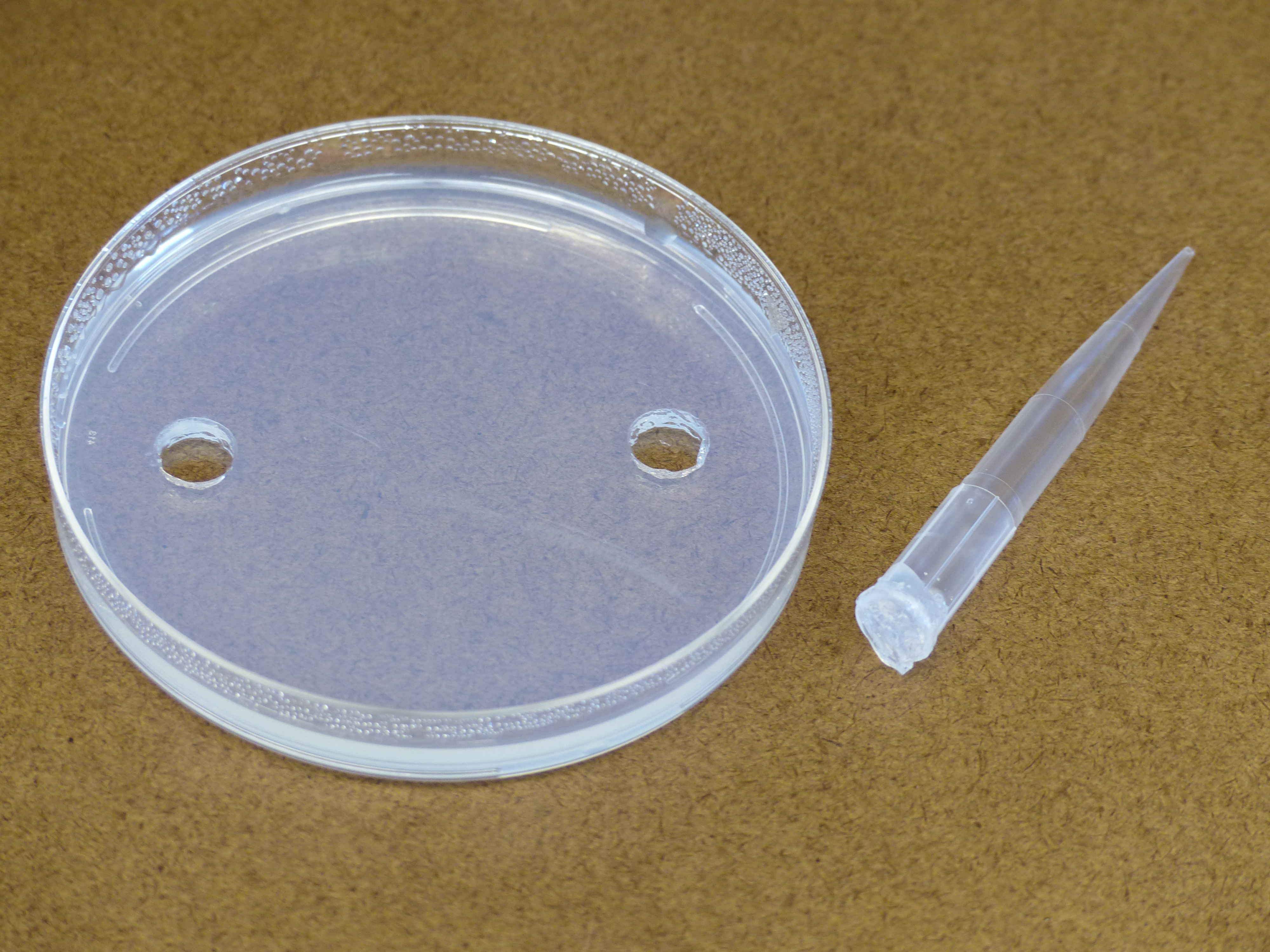


Step 3: Fill each core with YPD agar media.


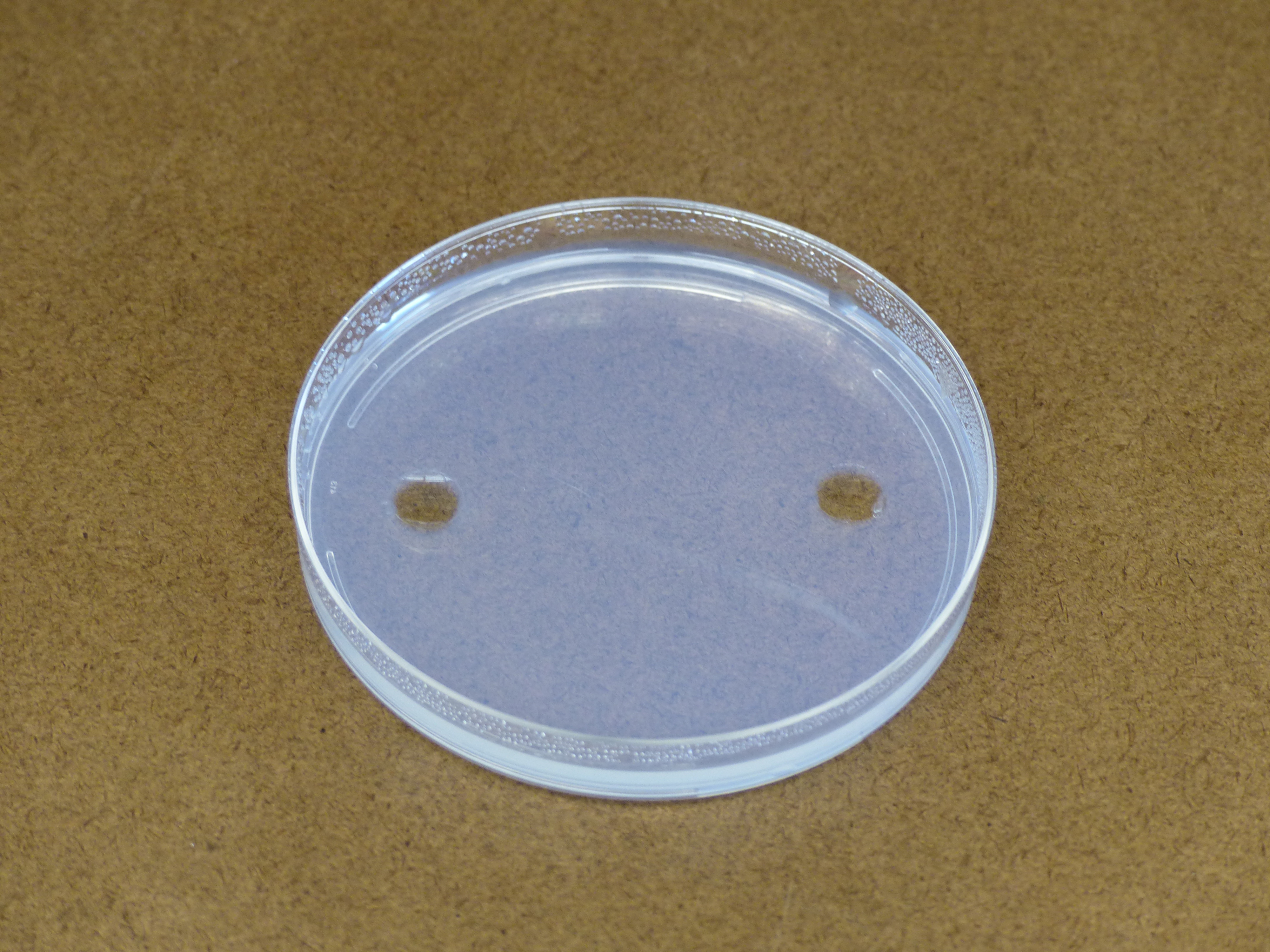


Step 4: Place actively growing yeast on top of each YPD core.
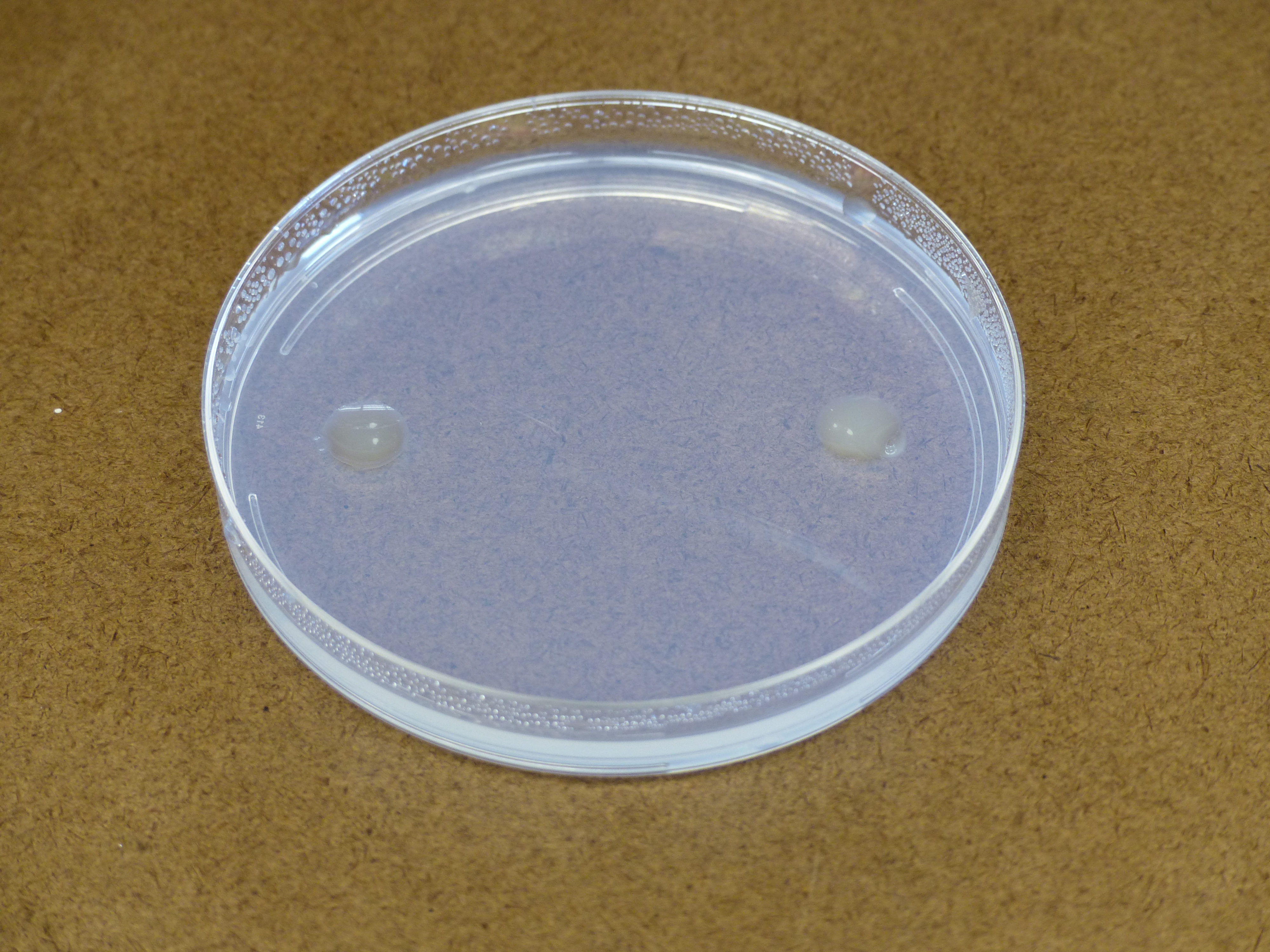


Step 5: 48 hours later, add 8 µL of either red or blue food dye to the yeast.
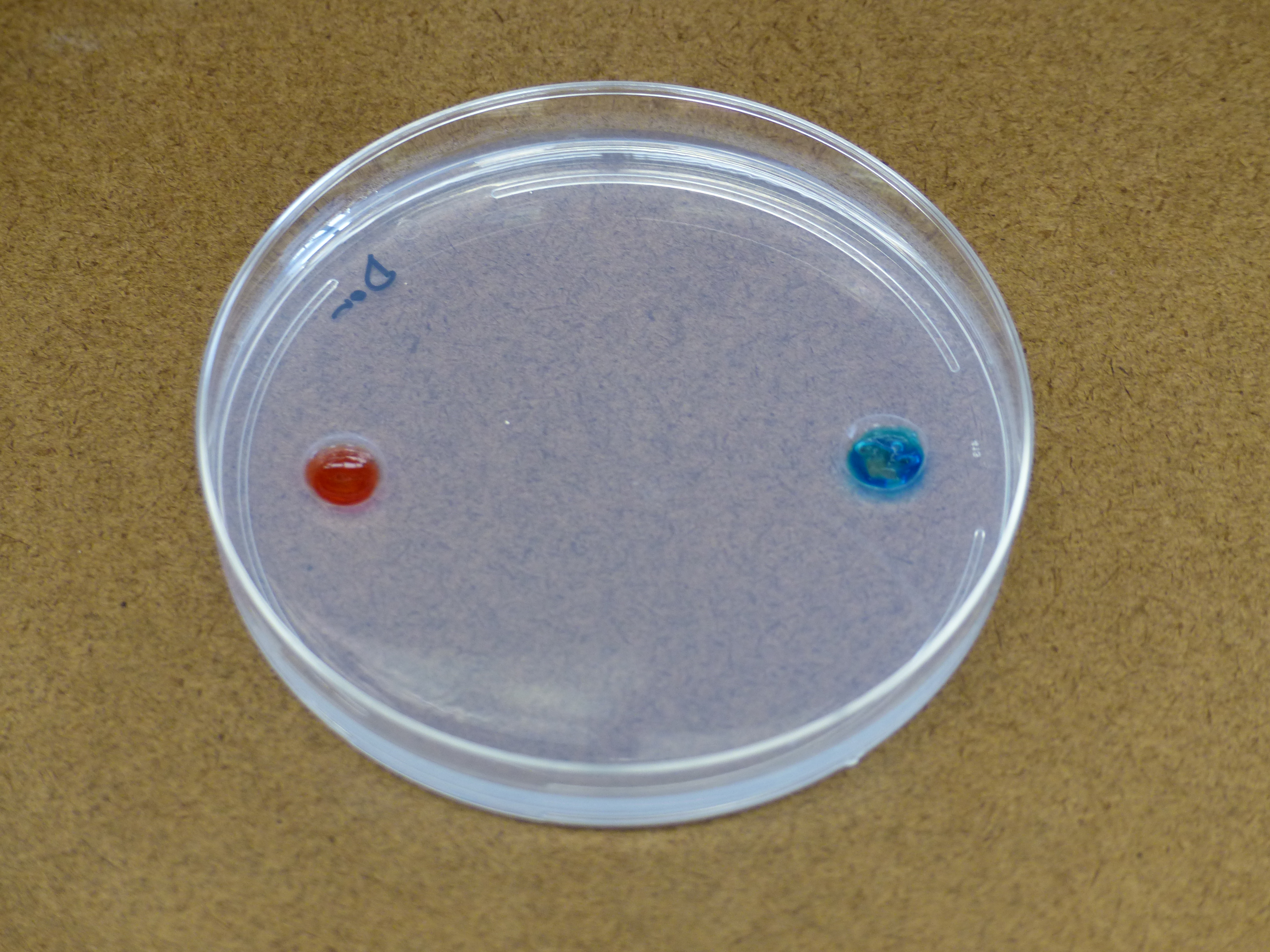


The final plates will look as follows: SC = *S. cerevisiae,* TY= Test yeast (for example *H. uvarum* or *S. paradoxus*), (Figure on following page).
